# Role of the NT-proBNP level in the diagnosis of pediatric heart failure and investigation of novel combined diagnostic criteria

**DOI:** 10.3892/etm.2013.1250

**Published:** 2013-08-05

**Authors:** CHUN-WANG LIN, XIANG-LIN ZENG, SHAO-HU JIANG, TONG WU, JIANG-PING WANG, JIN-FENG ZHANG, YANG-HUI OU

**Affiliations:** Department of Pediatrics, The Women and Children’s Health Care Hospital of Shunde, Shunde, Foshan, Guangdong 528300, P.R. China

**Keywords:** heart failure, amino-terminal pro-B-type natriuretic peptide, diagnostic criteria, children

## Abstract

The aim of this study was to investigate the modified Ross criteria score and the diagnostic cut-off level for plasmatic amino-terminal pro-brain natriuretic peptide (NT-proBNP) in the diagnosis of pediatric heart failure, by analyzing the receiver operating characteristic (ROC) curve. The plasma NT-proBNP level was measured in 80 children diagnosed with heart failure according to the modified Ross criteria, 80 children with non-cardiogenic dyspnea and 80 healthy children. The NT-proBNP levels were then compared using an F-test. The cut-off score for heart failure in the modified Ross criteria and the diagnostic cut-off level for plasmatic NT-proBNP in pediatric heart failure were determined by ROC curve analysis. The results demonstrated that the NT-proBNP level was markedly increased in 76 of the 80 children with heart failure, and the correlation with the modified Ross criteria was 95%. Based on ROC curve analysis, the diagnosis of pediatric heart failure was most accurate when the modified Ross criteria score was ≥4 and the plasmatic NT-proBNP level was ≥598 ng/l. The NT-proBNP level was normal (0–300 ng/l) in the children with non-cardiogenic dyspnea and the healthy children. Significant differences were observed in the comparison of the three groups (P<0.01). In conclusion, a NT-proBNP level of ≥598 ng/l, combined with a modified Ross criteria score ≥4, is highly diagnostic of heart failure in children.

## Introduction

Heart failure is one of the most serious diseases in children, and is characterized by a progressive left ventricular pump dysfunction, leading to cardiac dilatation, thinned walls and poor contractility (1). Following numerous investigations, pediatric heart failure has been redefined as a progressive clinical and pathophysiological syndrome that occurs due to cardiovascular and non-cardiovascular abnormalities. These abnormalities result in the characteristic signs and symptoms of the disease, including edema, respiratory distress, growth failure and exercise intolerance, accompanied by circulatory, neurohormonal and molecular disturbances. Cardiomyopathies and congenital heart disease are the most common causes, and lead to acute and chronic heart failure, respectively (2–4).

The diagnostic measures of pediatric heart failure include the Ross criteria (5), modified Ross criteria (6,7) and the New York University Pediatric Heart Failure Index (NYU PHFI) (8). Amino-terminal pro-brain natriuretic peptide (NT-proBNP) is a sensitive biomarker of heart failure, and is able to improve the accuracy of diagnosis (9–12). Serum NT-proBNP levels may be used to assist in the differentiation between dyspnea resulting from respiratory problems and heart failure (13), and have been demonstrated to correlate with the severity of left ventricular (LV) dysfunction and functional status (14). In addition, serum NT-proBNP levels have been revealed to be predictive of morbidity and mortality (15), and may be used in the selection of the therapeutic modality (16). Despite several studies demonstrating the significance of NT-proBNP as a biomarker for heart failure, its measurement is not commonly involved in the routine testing performed in children with cardiac disease, due to the fact that little is known about its function, accuracy and validity as a diagnostic test in children (17). The purpose of the current study was to determine whether plasma NT-proBNP levels in combination with a modified Ross criteria score constitute suitable diagnostic criteria for heart failure in children.

## Materials and methods

### Study population

A total of 240 children were involved in this study. These included 80 children with heart failure, who were admitted to the Women and Children’s Health Care Hospital of Shunde (Shunde, China) from March 2010 to January 2012, 80 children with non-cardiogenic dyspnea and 80 healthy children. The diagnosis of pediatric heart failure was made based on the modified Ross criteria (6,7). Among the 80 children with heart failure there were 43 males and 37 females, and the average age was 9.5 months (range, 1–18 months). Out of these 80, 36 had acute and 44 had chronic heart failure. Acute heart failure was caused by pneumonia in 22 children, shock in eight, myocarditis in two, cardiomyopathy in two, pericardial effusion in one and left atrial tumor complicated by cardiac tamponade syndrome in one. Chronic heart failure was due to a variety of congenital heart diseases, including ventricular septal defect in 22 children, atrial septal defect in nine, Fallot’s syndrome in five, aortic stenosis in two, patent ductus arteriosus in four and single-ventricle defects in two. Among the 80 children with non-cardiogenic dyspnea there were 43 males and 37 females, and the average age was 10.5 months (range, 1–20 months). The causes of the non-cardiogenic dyspnea were pneumonia in 73 children, chronic anemia in four, pleural effusion in two and congenital spina bifida combined with bulbar myelitis in one. The 80 healthy children included 40 males and 40 females, with an average age of 8.5 months (range, 1–16 months). The three groups had no significant differences with respect to age or gender (P>0.05 for each). Informed consent was obtained from each patient. The study was approved by the Ethics Committee of the The Women and Children’s Health Care Hospital of Shunde, Shunde, China.

### NT-ProBNP level assay

Prior to and following treatment, 2 ml whole blood was obtained from each child and stored in a fluid tube containing EDTA. Following this, 0.75 ml blood was aliquoted to a tube containing NT-proBNP quantitative buffer, and was mixed thoroughly. The NT-proBNP level was assayed within 5 min by the Canadian RAMP heart failure diagnostic instrument method. The testing materials were provided by the Response Biomedical Corp. (Vancouver, BC, Canada). The normal adult range of NT-proBNP is 0–300 ng/l, and a value of ≥450 ng/l is suggestive of heart failure. At present, there are no clear standards and there is no normal range for children.

### Data analysis

A receiver operating characteristic (ROC) curve was created, and the cut-off score for heart failure, based on the modified Ross criteria, and the diagnostic cut-off level for plasma NT-proBNP in pediatric heart failure were determined by analyzing the specificity and sensitivity of the diagnosis. The number of children with an elevated NT-ProBNP level in the heart failure group was compared with the number diagnosed with heart failure according to the modified Ross criteria. A coincidence rate was obtained by using the following formula: (N_high NT-ProBNP_/N_heart failure_) × 100. The data were analyzed using the F-test, and statistical analyses were performed using SPSS version 13.0 statistical software (SPSS, Inc., Chicago, IL, USA). P< 0.05 was considered to indicate a statistically significant difference.

## Results

### Analysis of the cut-off score for heart failure in the modified Ross criteria

**Figure 1.**
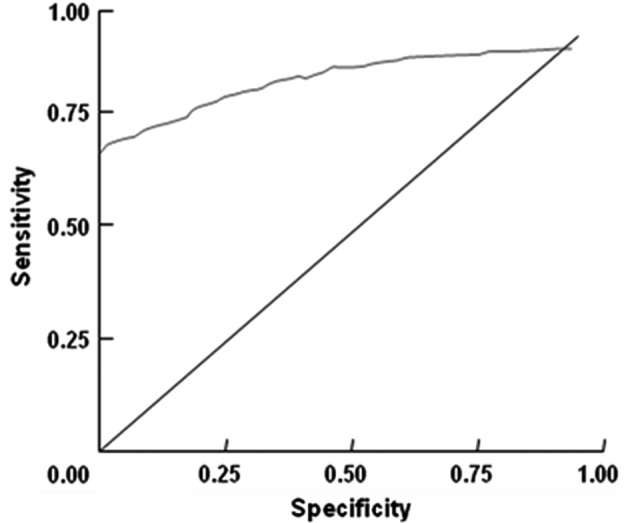
Receiver operating characteristic (ROC) curve of heart failure based on the modified Ross criteria.

The ROC area under the curve (AUC) of heart failure in the modified Ross criteria was 0.958 [95% confidence interval (CI), 0.942–0.993] (Fig. 1). A diagnostic score of ≥4, according to the ROC curve, exhibited the highest sensitivity and specificity for heart failure (Table I). However, it was observed that the specificity and sensitivity were relatively low when the diagnostic cut-off was ≥3 in the Ross criteria, which readily led to misdiagnosis.

### Analysis of the diagnostic cut-off level for plasma NT-proBNP in pediatric heart failure

**Figure 2.**
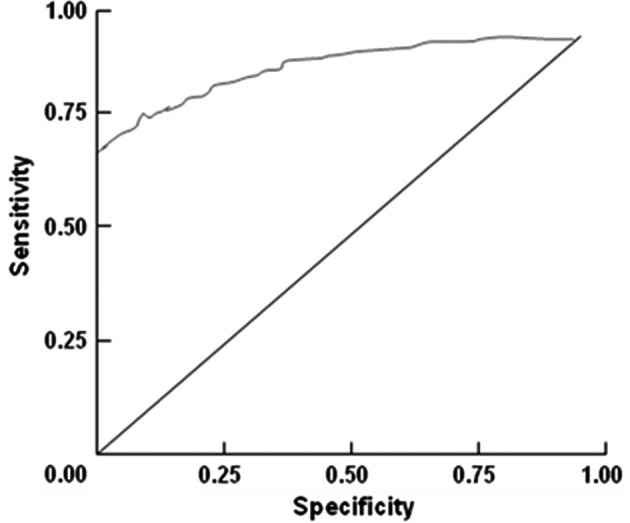
Receiver operating characteristic (ROC) curve of the plasma amino-terminal pro-brain natriuretic peptide (NT-proBNP) level in pediatric heart failure.

The ROC AUC of plasma NT-proBNP level in pediatric heart failure was 0.979 (95% CI, 0.948–0.994; Fig. 2). Based on the ROC curve, a value of ≥598 ng/l was selected as the diagnostic cut-off, as it exhibited a high sensitivity and specificity for a diagnosis of pediatric heart failure.

### Correlation between a high NT-proBNP level and a diagnosis of heart failure, according to the modified Ross criteria

It was observed that the NT-ProBNP level in 76 of the 80 children in the heart failure group was >598 ng/l. The correlation with the modified Ross criteria was 95%.

### Correlation analysis between the NT-proBNP level in mild, moderate or severe heart failure and the modified Ross criteria

The NT-proBNP levels used in the present study to assess mild (41 cases), moderate (27 cases) and severe heart failure (12 cases), which were diagnosed according to the modified Ross criteria, were 1,184.31 (95% CI, 716.61–2,935.35), 3,353.57 (95% CI, 3,008.83–3,699.25) and 16,883.22 (95% CI, 5,718.77–5,000.32 ng/l), respectively. There was no overlap in the 95% CIs (Table II).

The F-test revealed that there were significant differences in the NT-proBNP levels among the mild, moderate and severe heart failure groups [F=29.74, P=0.000 (P<0.001)] (Table III). The plasma NT-proBNP level increased with increasing severity of heart failure [correlation coefficient r= 0.675, P=0.000 (P<0.001)].

### Comparison between the NT-proBNP level in the heart failure, non-cardiogenic dyspnea and healthy groups

The F-test revealed that the NT-ProBNP levels in the group with heart failure, the group with non-cardiogenic dyspnea and the group of healthy children were significantly different [F=14.90, P=0.000 (P<0.001)] (Table III). The NT-ProBNP level in the heart failure group was significantly higher than that in the other two groups (P= 0.001 for each). There was no significant difference between the NT-ProBNP levels of the group with non-cardiogenic dyspnea and the group of healthy children (P=0.097).

### Combined diagnostic criteria for pediatric heart failure

The results of the study indicate that the NT-ProBNP level is a sensitive and reliable biomarker for the diagnosis of heart failure, and is highly correlated with the modified Ross criteria. Analysis of the ROC curve revealed that a score of ≥4 in the modified Ross criteria was notably more effective than a score of ≥3 for the diagnosis of pediatric heart failure. Furthermore, the accuracy of the diagnosis greatly improved when the plasma NT-proBNP level was set at ≥598 ng/l. Based on these data, we designed combined diagnostic criteria for heart failure (Table IV) and suggest that the score ranges for non-heart failure and mild, moderate and severe heart failure be set at 0–4, 5–8, 9–12, and 13–15, respectively.

## Discussion

The importance of the NT-proBNP level in the diagnosis and evaluation of heart failure has been widely accepted (9–11). BNP acts on several organs and facilitates sodium transfer, increases urine production, dilates vessels and inhibits the renin-angiotensin-aldosterone system and sympathetic nerves. The NT-proBNP level has been observed to increase with the abnormally high intraventricular pressure that is associated with heart failure (9), and the level is positively correlated with the severity of heart failure. The possibility of heart failure is low when the NT-proBNP level is <400 ng/l, with a negative predictive value of ∼90%. However, heart failure is likely when the level is >450 ng/l, when the positive predictive value is also ∼90% (12). The results of the current study indicate that a NT-proBNP level >598 ng/l was predictive of a diagnosis of pediatric heart failure. In the current study, an increased NT-proBNP level was present in 95% of the 80 children who were diagnosed with heart failure according to the modified Ross criteria. Moreover, there were also significant differences in the NT-proBNP levels among cases of mild, moderate and severe heart failure.

The commonly utilized criteria of heart failure include the international Ross criteria, the modified Ross criteria and the NYU PHFI score. All of these, with the exception of the NYU PHFI score, are based on clinical signs and symptoms. In 1987, based on the classification of heart failure for adults, designed by the New York Heart Association, Ross proposed a novel pediatric classification for babies aged <6 months (5). This was subsequently modified by Reithmann *et al* (6) and Läer *et al* (7) to be suitable for children between 0 and 14 years of age, and is currently widely used in the diagnosis of pediatric heart failure (18). Although the modified Ross criteria are widely applied, they are based on clinical symptoms and signs, and possess a certain degree of subjectivity. Therefore, the diagnosis is likely to have an enhanced accuracy and sensitivity with the addition of an objective diagnostic parameter to the criteria. It was observed in the present study that the rate of misdiagnosis was high when a modified Ross criteria score of ≥3 was used as the diagnostic cut-off. By analyzing the specificity and sensitivity of the ROC curve, it was revealed that a score of ≥4 demonstrated an enhanced efficacy for the diagnosis of pediatric heart failure.

Aside from the score parameters in the modified Ross criteria, the NYU PHFI score (8) also includes the results of echocardiography and chest radiography examinations, and scores for drug therapy. Although the NYU PHFI score is more sensitive and specific than the modified Ross criteria, it is not commonly used. This is because the score involves a large number of parameters, and certain parameters are particularly complicated.

The combined diagnostic criteria for heart failure proposed in the present study were based on the modified Ross criteria, and introduced an objective diagnostic parameter, the NT-proBNP level. The results of this study demonstrated that the diagnosis of pediatric heart failure according to the modified Ross criteria and the NT-proBNP level was 95% accurate. It was observed that ∼93% of children with mild and 100% of children with severe heart failure were included in the 95% CI. In addition, there was no overlap in the 95% CIs of mild, moderate and severe heart failure, and the NT-proBNP level was observed to increase with increasing modified Ross criteria scores. Therefore, the modified Ross criteria and the NT-proBNP level were included in the combined diagnostic criteria for heart failure, in order to achieve a greater accuracy of diagnosis. Moreover, the measurement of the NT-proBNP level is easy, fast, accurate and inexpensive.

## Figures and Tables

**Table I.** Sensitivity and specificity of heart failure diagnosis based on different Ross scores.

Ross score	Sensitivity	Specificity	Sensitivity + specificity	Sensitivity + specificity −1
0.0	1.000	0.000	1.000	0.000
2.0	0.823	0.625	1.448	0.448
3.0	0.886	0.798	1.684	0.684
4.0	0.908	0.952	1.860	0.860
5.0	0.902	0.957	1.819	0.819
6.0	0.906	0.936	1.842	0.842
7.0	0.802	1.000	1.802	0.802
8.0	0.887	0.958	1.845	0.845

**Table II.** Comparison of plasma NT-proBNP levels and modified Ross criteria scores in mild, moderate and severe heart failure.

Heart failure	n	NT-ProBNP level (ng/l)			
		406.98–576.05	614.38–2935.35	3004.17–3699.25	5718.77–35000.32
Mild	41	491.52 (4)	1774.87 (37)	0 (0)	0 (0)
Moderate	27	0 (0)	0 (0)	3351.71 (27)	0 (0)
Severe	12	0 (0)	0 (0)	0 (0)	20359.55 (12)

F=30.16, P=0.000 (P<0.001). Results are presented as the median score (number of cases). NT-proBNP, amino-terminal pro-brain natriuretic peptide.

**Table III.** Median amino-terminal pro-brain natriuretic peptide (NT-proBNP) levels in the three study groups.

Group	n	NT-proBNP level (ng/l)	F-statistic	P-value
Heart failure	80	17703.65	-	-
Non-cardiogenic dyspnea	80	210.23	-	-
Healthy	80	214.27	13.65	0.000

**Table IV.** Scoring method for a novel combined diagnostic criteria for heart failure.

Item	0 score	+1 score	+2 score	+3 score
Sweating	Head only	Head and torso during activities	Head and torso at rest	
Shortness of breath	Absent	Present during activities	Present at rest	
Concave disorder	Absent	Present-mild	Present-severe	
Respiration rate (breaths/min)				
0–1 years of age	<50	50–60	>60	
2–6 years of age	<35	35–45	>45	
7–10 years of age	<25	25–35	>35	
11–14 years of age	<18	18–28	>28	
Heart rate (beats/min)				
0–1 years of age	<160	160–170	>170	
2–6 years of age	<105	105–115	>115	
7–10 years of age	<90	90–100	>100	
11–14 years of age	<80	80–90	>90	
Liver enlargement (cm)	<2	2–3	>3	
NT-ProBNP index	<598	598–3000	>3000–5000	>5000

Scores for heart failure using the modified Ross criteria (without NT-proBNP index): No heart failure, 0–2; mild heart failure, 3–6; moderate heart failure, 7–9 and severe heart failure, 10–12. Scores with the novel combined diagnostic criteria: No heart failure, 0–4; mild heart failure, 5–8; moderate heart failure, 9–12 and severe heart failure, 13–15. NT-proBNP, amino-terminal pro-brain natriuretic peptide.
